# Transcriptomic Characterization Reveals Mitochondrial Involvement in Nrf2/Keap1-Mediated Osteoclastogenesis

**DOI:** 10.3390/antiox13121575

**Published:** 2024-12-20

**Authors:** Eiko Sakai, Takayuki Tsukuba

**Affiliations:** Department of Dental Pharmacology, Graduate School of Biomedical Sciences, Nagasaki University, 1-7-1, Sakamoto, Nagasaki 852-8588, Japan; tsuta@nagasaki-u.ac.jp

**Keywords:** osteoclast, oxidative stress, Nrf2, Keap1, transcriptome, gene ontology, GeneMANIA

## Abstract

Although osteoclasts play crucial roles in the skeletal system, the mechanisms that underlie oxidative stress during osteoclastogenesis remain unclear. The transcription factor Nrf2 and its suppressor, Keap1, function as central mediators of oxidative stress. To further elucidate the function of Nrf2/Keap1-mediated oxidative stress regulation in osteoclastogenesis, DNA microarray analysis was conducted in this study using wild-type (WT), *Keap1* knockout (*Keap1* KO), and *Nrf2* knockout (*Nrf2* KO) osteoclasts. Principal component analysis showed that 403 genes, including *Nqo1*, *Il1f9*, and *Mmp12*, were upregulated in *Keap1* KO compared with WT osteoclasts, whereas 24 genes, including *Snhg6*, *Ccdc109b*, and *Wfdc17*, were upregulated in *Nrf2* KO compared with WT osteoclasts. Moreover, 683 genes, including *Car2*, *Calcr*, and *Pate4*, were upregulated in *Nrf2* KO cells compared to *Keap1* KO cells. Functional analysis by Gene Ontology and Kyoto Encyclopedia of Genes and Genomes pathway analysis showed upregulated genes in *Nrf2* KO osteoclasts were mostly enriched in oxidative phosphorylation. Furthermore, GeneMANIA predicted the protein–protein interaction network of novel molecules such as Rufy4 from genes upregulated in *Nrf2* KO osteoclasts. Understanding the complex interactions between these molecules may pave the way for developing promising therapeutic strategies against bone metabolic diseases caused by increased osteoclast differentiation under oxidative stress.

## 1. Introduction

Osteoclasts are multinucleated cells responsible for bone resorption and play crucial roles in physiological bone remodeling, pathological osteoporosis, rheumatoid arthritis, and periodontal diseases [1,2,3]. Physiologically, the receptor activator of nuclear factor-kappa B ligand (RANKL) promotes osteoclast differentiation. Nuclear factor of activated T cells c1 (NFATc1) is an important transcription factor that promotes osteoclastogenesis [4]. Inflammatory cytokines such as interleukin 1 (IL-1), IL-6, and TNF-α [5], as well as lipopolysaccharides (LPS) and oxidative stress [6], promote osteoclast differentiation. In an in vitro culture system using bone marrow cells from adult mice, osteoclasts matured in monocyte-macrophage lineage-derived progenitor cells grown with macrophage colony-stimulating factor (M-CSF) when RANKL was added [7]. During osteoclast differentiation, there is an increase in the expression of carbonic anhydrase 2, calcitonin receptor, vacuolar ATPase, cathepsin K, Ocstamp, Dcstamp [3], Oscar [8], Steap4 [9], and Rab38 [10].

Nuclear factor erythroid 2-related factor 2 (Nrf2) is a crucial transcription factor involved in antioxidant responses [11]. Physiologically, Nrf2 binds to its inhibitor, Keap1, promoting its degradation in a proteasome-dependent manner, maintaining low intracellular levels. Under oxidative stress, Keap1 is released from Nrf2, and Nrf2 translocates to the nucleus to upregulate cytoprotective genes [12,13]. A previous study using *Nrf2*-specific siRNA probes and *Keap1* and *Nrf2* expression plasmids demonstrated that the Keap1/Nrf2 axis regulates RANKL-dependent osteoclast differentiation [14]. Moreover, natural compounds with Nrf2-activating ability, such as epigallocatechin gallate [15], fisetin [16], resveratrol [17], and sulforaphane [15,18], inhibit osteoclast differentiation.

Studies using bone marrow cells from *Nrf2* knockout (*Nrf2* KO) mice have reported enhanced osteoclast differentiation [19,20]. Because *Keap1* knockout (*Keap1* KO) mice with Nrf2 hyperactivation are juvenile lethal [13], we previously used splenocytes from newborn mice instead of bone marrow cells as osteoclast progenitor cells, cultured them with M-CSF, and stimulated the cells with RANKL to determine whether osteoclast differentiation occurred. We observed that osteoclast differentiation was markedly suppressed in Keap1 KO cells [21], which is particularly relevant to the finding that many Nrf2-activating natural compounds suppress osteoclast differentiation [22]. However, the mechanisms underlying this suppression are not fully understood.

Microarray analysis is a robust technique for identifying the genes and pathways involved in biological processes in an unbiased manner. In this study, we used gene array technology to identify changes in gene expression during the differentiation of mouse splenocyte-derived macrophages into osteoclasts. We analyzed the transcriptome of RANKL-stimulated wild-type (WT), *Nrf2* KO, and *Keap1* KO cells to identify novel osteoclast regulators and elucidate the mechanisms underlying osteoclast differentiation via the Nrf2/Keap1 system.

Here, we report all transcripts discovered in a microarray comparison of highly purified WT, *Keap1* KO, and *Nrf2* KO osteoclasts stimulated with RANKL. When compared with Keap1 KO cells, Nrf2 KO osteoclasts generally showed higher expression of osteoclast marker genes such as *Oscar*, *Calcr*, *Mmp9*, *Acp5*, *Ctsk*, and *Car2*. Apart from the above-mentioned osteoclastogenic genes, whose expression is upregulated by *Nrf2* KO, the relationship of the following mitochondria-related genes, including *Ndhfs*, *Cox*, and *Sdhc*, as well as novel genes, with osteoclastogenesis is not yet clear.

## 2. Materials and Methods

### 2.1. Reagents

RANKL was prepared according to a previously described method using an expression vector containing the hexahistidine-tagged human soluble RANKL extracellular domain, kindly provided by Dr. H. Amano (Tokyo Medical and Dental University, Tokyo, Japan) [23]. In brief, Codon-Plus BL21 (DE3) RL-competent cells were used to express recombinant RANKL, which was then purified using Ni-NTA column chromatography. Contaminated LPS was removed by phase separation using Triton X-114, resulting in a final LPS concentration below the detection limit (1 pg/μg protein). In an osteoclastogenesis assay using mouse bone marrow cells, the purified recombinant RANKL protein demonstrated bioactivity comparable to human soluble RANKL (PeproTech EC, London, UK).

### 2.2. Mice

Breeding pairs of *Nrf2*^−/−^ mice (*Nrf2* KO) (RBRC01390) [24] were obtained by RIKEN BRC through the National Bio-Resource Project of MEXT, Japan (Tsukuba, Japan). *Keap1*^+/−^ (RBRC01388) [13] were provided by RIKEN BRC and were self-mated to generate *Keap1* wild-type (WT) control mice and *Keap1*^−/−^ homozygous mice (*Keap1* KO). The Animal Care and Use Committee of Nagasaki University Graduate School of Biomedical Sciences approved all animal experimental protocols (Approval Number: 210216169-2). All the experiments were conducted in accordance with the rules of animal experimentation at Nagasaki University, Japan.

### 2.3. Cell Culture

Osteoclast precursors obtained from the spleen of neonatal mice were used for DNA microarray analysis as previously described [21]. In brief, splenocytes were cultured in α-minimal essential medium supplemented with 10% fetal bovine serum, penicillin (100 U/mL), streptomycin (100 μg/mL), and amphotericin B (0.25 μg/mL) in the presence of macrophage colony-stimulating factor (M-CSF; 50 ng/mL) for 16 h at 37 °C under 5% CO_2_. Non-adherent cells were collected and cultured in M-CSF (50 ng/mL). After 72 h, the adherent splenic macrophages were cultured for three days with M-CSF (30 ng/mL) and RANKL (50 ng/mL) to produce osteoclasts. As previously described [25], the cells were fixed with 4% paraformaldehyde on day three and stained for tartrate-resistant acid phosphatase (TRAP) activity to verify osteoclast formation. For bone marrow macrophage (BMM)-derived osteoclast formation, marrow cells from the femurs and tibias of 5-week-old male *Nrf2* KO and WT mice were cultured using the method described above, in which osteoclasts were prepared from splenocytes.

### 2.4. Microarray Analysis

Three days after the addition of RANKL, total RNA was extracted from WT, *Nrf2* KO, and *Keap1* KO cells using TRIzol Reagent (Thermo Fisher Scientific, Waltham, MA, USA) and subsequently purified using an RNeasy Mini Kit (QIAGEN, Tokyo, Japan) according to the manufacturer’s instructions. The RNA quality was evaluated using an Agilent 2100 Bioanalyzer (Agilent Technologies, Palo Alto, CA, USA) and a NanoDrop spectrophotometer (Thermo Fisher). The values of 260/280 were more than 2.00 for all samples. Total RNA (100 ng) was reverse transcribed using an Affymetrix GeneChip WT Plus Reagent Kit (Affymetrix, Santa Clara, CA, USA), labeled with biotin using a GeneChip WT Terminal Labeling Kit (Affymetrix), hybridized to a GeneChip Mouse Gene 2.0 ST Array (Affymetrix) using a GeneChip Hybridization, Wash, and Stain Kit (Affymetrix), and scanned according to the manufacturer’s instructions. Numerical data files of the three arrays of WT, *Nrf2* KO, and *Keap1* KO mice (CHP files obtained by the Robust Multichip Analysis algorithm on the Affymetrix Expression Console) were compared and analyzed using GeneSpring GX analysis software (version 7.3.1, Agilent Technologies, Palo Alto, CA, USA). For functional enrichment analysis, gene ontology (GO) analysis was performed using the Database for Annotation, Visualization, and Integrated Discovery (DAVID; https://david.ncifcrf.gov/, accessed on 24 October 2024), and pathway analysis was performed using the Kyoto Encyclopedia of Genes and Genomes (KEGG). Protein–protein interaction networks were predicted using GeneMANIA (https://genemania.org/, accessed on 24 October 2024). Probes that had values of fold change (log) > 1 or (log) < −1 between the samples to be compared were considered statistically significant.

### 2.5. Quantitative Real-Time Polymerase Chain Reaction (RT-PCR) Analysis

To verify the microarray data, Quantitative RT-PCR (qRT-PCR) analysis was performed using Quantstudio 3 (Thermo Fisher Scientific) according to the manufacturer’s instructions. Briefly, total RNA was extracted with TRIzol Reagent and reverse transcription was performed using oligo(dT)15 primer (Promega, Madison, WI, USA) and Revertra Ace (Toyobo, Osaka, Japan). Complementary DNA was amplified using Brilliant III Ultra-Fast SYBR Green QPCR Master Mix (Agilent Technologies). The primer sets used are listed in Supplementary Table S1. All samples were assayed in triplicate, and the relative mRNA expression was normalized to that of the housekeeping gene *β-actin*. Data are presented as the mean ± standard deviation (SD) from three independent experiments. Statistical significance was determined using Student’s *t*-test, and differences were considered statistically significant at * *p* < 0.05 and ** *p* < 0.01.

## 3. Results

### 3.1. Identification of Differentially Expressed Genes

**Figure 1:**
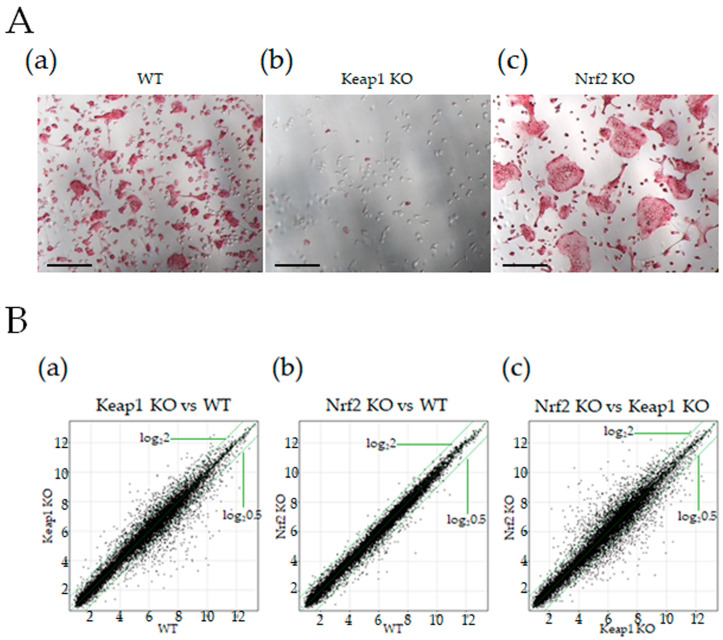
Microarray analysis of WT, *Nrf2* KO, and *Keap1* KO cells. (**A**) Splenic macrophages from WT, *Keap1* KO, and *Nrf2* KO mice were cultured with 30 ng/mL M-CSF and 50 ng/mL RANKL for three days, followed by TRAP staining. Representative photographs showing red-colored osteoclasts. (**a**) WT, (**b**) *Keap1* KO, and (**c**) *Nrf2* KO mice. Scale bars: 100 μm. (**B**) Splenic macrophages from two mice each of WT, *Keap1* KO, and *Nrf2* KO were cultured with 30 ng/mL M-CSF and 50 ng/mL RANKL for three days, and RNA was collected from each cell for DNA microarray analysis (single microarray analysis for each cell). Graphs showing scatter plots of (**a**) *Keap1* KO cells vs. WT osteoclasts, (**b**) *Nrf2* KO osteoclasts vs. WT osteoclasts, and (**c**) *Nrf2* KO osteoclasts vs. *Keap1* KO cells. Green lines indicate log_2_2 or log_2_0.5.

We previously demonstrated that in the presence of RANKL, *Keap1* KO splenic macrophages failed to differentiate into osteoclasts. However, compared to WT cells, *Nrf2* KO splenic macrophages produced significantly higher osteoclasts (Figure 1A). To elucidate the role of the Nrf2/Keap1 system during osteoclast differentiation, we performed a comparative analysis of gene expression in the following three combinations to identify the genes that fluctuate in relation to the system.

1. *Keap1* KO vs. WT;
2. *Nrf2* KO vs. WT;
3. *Nrf2* KO vs. *Keap1* KO.

Principal component analysis (PCA) results showed that the gene expression patterns in *Keap1* KO cells differed from those in WT and *Nrf2* KO osteoclasts, whereas the expression patterns in *Nrf2* KO osteoclasts were similar to those in WT osteoclasts. This may be because *Keap1* KO cells did not form osteoclasts, unlike WT and *Nrf2* KO cells (Figure 1B).

**Figure 2:**
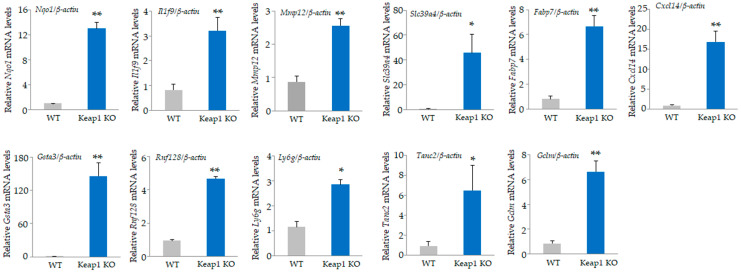
Validation of microarray data. Upregulated genes in Table 1 were confirmed by qRT-PCR. The relative mRNA levels of *Nqo1*, *Il1f9*, *Mmp12*, *Slc39a4*, *Fabp7*, *Cxcl14*, *Gsta3*, *Rnf128*, *Ly6g*, *Tanc2*, and *Gclm* in *Keap1* KO were confirmed. Data are presented as the mean ± SD from three independent experiments (* *p* < 0.05 and ** *p* < 0.01).

**Figure 3:**
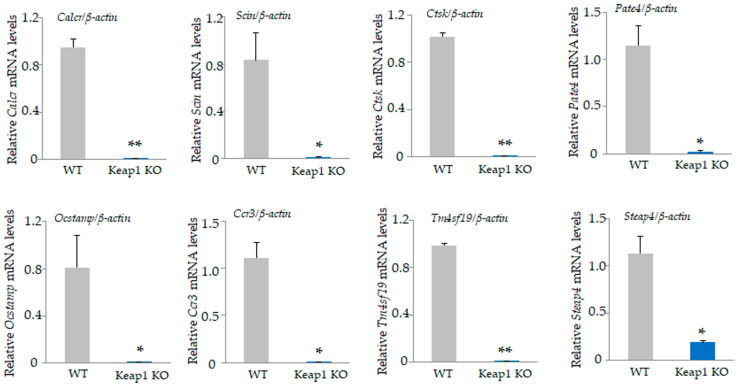
Validation of microarray data. Downregulated genes in Table 2 were confirmed by qRT-PCR. The relative mRNA levels of *Calcr*, *Scin*, *Ctsk*, *Pate4*, *Ocstamp*, *Ccr3*, *Tm4sf19*, and *Steap4* in *Keap1* KO were confirmed. Data are presented as the mean ± SD from three independent experiments (* *p* < 0.05 and ** *p* < 0.01).

Among the 34,390 genes, 403 were upregulated by at least 2-fold in *Keap1* KO cells than in WT osteoclasts (Supplementary Table S2), and 553 were downregulated by less than 0.5-fold (Supplementary Table S3). The top 20 genes, including NAD(P)H dehydrogenase, quinone1 (*Nqo1*), interleukin 1 family member 9 (*Il1f9*), and matrix metallopeptidase 12 (*Mmp12*), and bottom 20 downregulated genes, including carbonic anhydrase 2 (*Car2*), calcitonin receptor (*Calcr*), and osteoclast-associated receptor (*Oscar*), are listed in Table 1 and Table 2. We validated the mRNA expression of these genes by qRT-PCR (Figure 2 and Figure 3). Consistent with the microarray results, significant upregulation of *Nqo1*, *Il1f9*, *Mmp12*, *Slc39a4*, *Fabp7*, *Cxcl14*, *Gsta3*, *Rnf128*, *Ly6g*, *Tanc2*, and *Gclm* was confirmed in *Keap1* KO compared to WT (Figure 2), while *Calcr*, *Scin*, *Ctsk*, *Pate4*, *Ocstamp*, *Ccr3*, *Tm4sf19*, and *Steap4* were significantly decreased (Figure 3).

**Figure 4:**
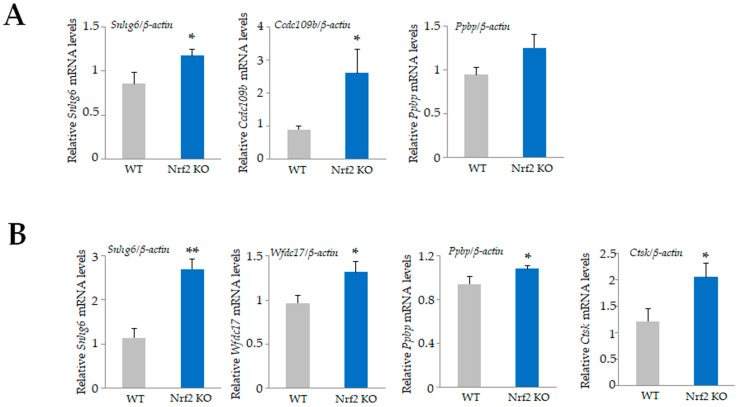
Validation of microarray data. Upregulated genes in Table 3 were confirmed by qRT-PCR. (**A**) Significant upregulation of *Snhg6* and *ccdc109b* in *Nrf2* KO osteoclasts derived from splenocyte were confirmed. *Ppbp* gene expression tended to increase. (**B**) Significant upregulation of *Snhg6*, *Wfdc17*, *Ppbp*, and *Ctsk* in *Nrf2* KO osteoclasts derived from BMMs were confirmed. Data are presented as the mean ± SD from three independent experiments (* *p* < 0.05 and ** *p* < 0.01).

**Figure 5:**
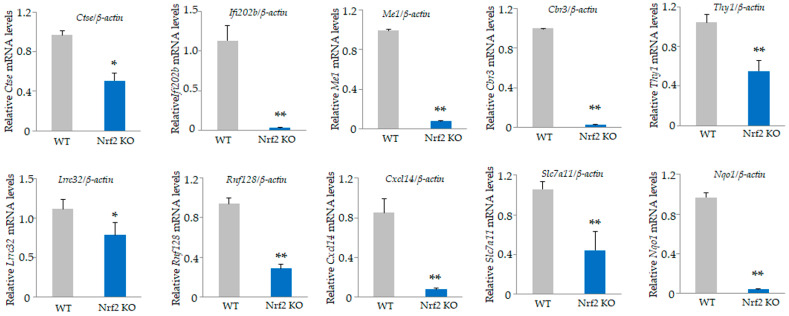
Validation of microarray data. Downregulated genes in Table 4 were confirmed by qRT-PCR. Significant downregulation of *Ctse*, *Ifi202b*, *Me1*, *Cbr3*, *Thy1*, *Lrrc32*, *Rnf128*, *Cxcl14*, *Slc7a11*, and *Nqo1* in *Nrf2* KO osteoclasts were confirmed. Data are presented as the mean ± SD from three independent experiments (* *p* < 0.05 and ** *p* < 0.01).

Among the 34,390 genes, 24 genes were upregulated by at least 2-fold (Supplementary Table S4) and 112 genes were downregulated in *Nrf2* KO osteoclasts compared with WT osteoclasts (Supplementary Table S5). The top 20 upregulated genes, including small nuclear RNA host gene 6 (*Snhg6*), coiled-coil domain containing 109 B (*Ccdc109b*), and WAP four-disulfide core domain 17 (*Wfdc17*), and the bottom 20 downregulated genes, including cathepsin E (*Ctse*), interferon activated gene 202 B (*Ifi202b*), malic enzyme 1, NADP(+)-dependent, and cytosolic (*Me1*), are listed in Table 3 and Table 4. We validated the mRNA expression of these genes by qRT-PCR (Figure 4 and Figure 5). The qRT-PCR results showed a significant upregulation of *Snhg6* and *Ccdc109b* genes in splenic macrophage-derived *Nrf2* KO osteoclasts (Figure 4A). Since *Nrf2* KO mice grow to adulthood, osteoclasts prepared from BMMs of 5-week-old mice similarly showed a significant increase in *Snhg6*, *Ppbp*, *Wfdc17*, and *Ctsk,* consistent with the microarray results (Figure 4B). Moreover, a significant decrease in *Ctse*, *Ifi202b*, *Me1*, *Cbr3*, *Thy1*, *Lrrc32*, *Rnf128*, *Cxcl14*, *Slc7a11*, and *Nqo1* were confirmed in splenic macrophage-derived *Nrf2* KO osteoclasts (Figure 5).

**Figure 6:**
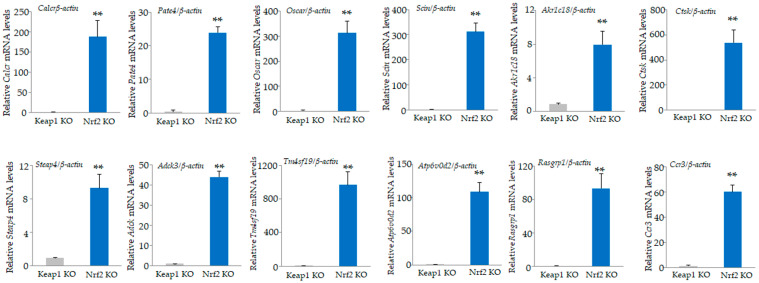
Validation of microarray data. Upregulated genes in Table 5 were confirmed by qRT-PCR. Significant upregulation of *Calcr*, *Pate4*, *Oscar*, *Scin*, *Akr1c18*, *Ctsk*, *Steap4*, *Adck3*, *Tm4sf19*, *Atp6v0d2*, and *Ccr3* in *Nrf2* KO osteoclasts were confirmed. Data are presented as the mean ± SD from three independent experiments (** *p* < 0.01).

**Figure 7:**
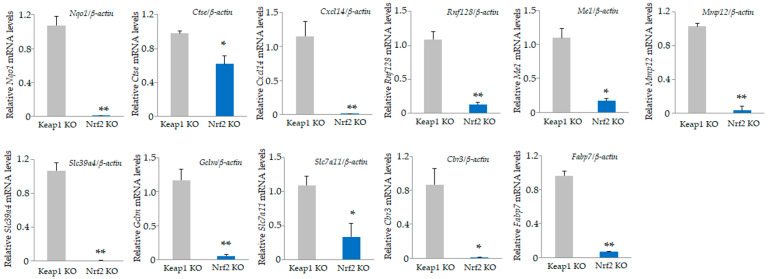
Validation of microarray data. Downregulated genes in Table 6 were confirmed by qRT-PCR. Significant downregulation of *Nqo1*, *Ctse*, *Cxcl14*, *Rnf128*, *Me1*, *Mmp12*, *Slc39a4*, *Gclm*, *Slc7a11*, *Cbr3*, and *Fabp7* in *Nrf2* KO osteoclasts were confirmed. Data are presented as the mean ± SD from three independent experiments (* *p* < 0.05 and ** *p* < 0.01).

Among the 34,390 genes, 683 were upregulated by at least 2-fold in *Nrf2* KO osteoclasts compared to *Keap1* KO cells (Supplementary Table S6), and 644 were downregulated in *Nrf2* KO osteoclasts compared with *Keap1* KO cells (Supplementary Table S7). The top 20 upregulated genes, including carbonic anhydrase 2 (*Car2*), calcitonin receptor (*Calcr*), and prostate and testis expressed 4 (*Pate4*); and the bottom 20 downregulated genes, including NAD(P)H dehydrogenase, quinone 1 (*Nqo1*), cathepsin E (*Ctse*), and chemokine (C-X-C motif) ligand 14 (*Cxcl14*), are listed in Table 5 and Table 6. We validated the mRNA expression of these genes by qRT-PCR (Figure 6 and Figure 7). Consistent with the microarray results, significant upregulation of *Calcr*, *Pate4*, *Oscar*, *Scin*, *Akr1c18*, *Ctsk*, *Steap4*, *Adck3*, *Tm4sf19*, *Atp6v0d2*, and *Ccr3* in *Nrf2* KO osteoclasts were confirmed (Figure 6), while significant downregulation of *Nqo1*, *Ctse*, *Cxcl14*, *Rnf128*, *Me1*, *Mmp12*, *Slc39a4*, *Gclm*, *Slc7a11*, *Cbr3*, and *Fabp7* in *Nrf2* KO osteoclasts were confirmed (Figure 7).

### 3.2. GO Analysis of Nrf2/Keap1-Mediated Osteoclastogenesis

**Figure 8:**
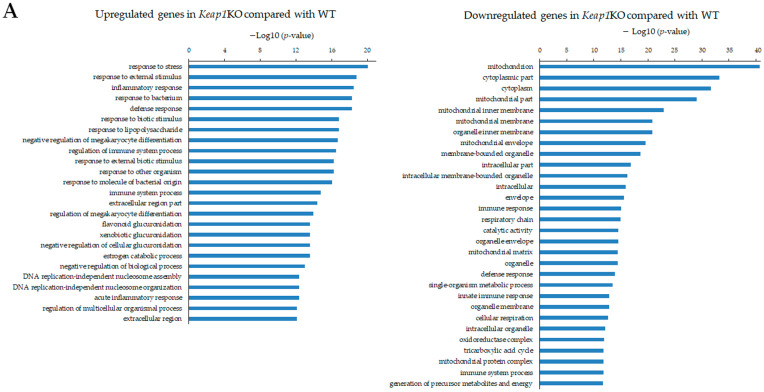

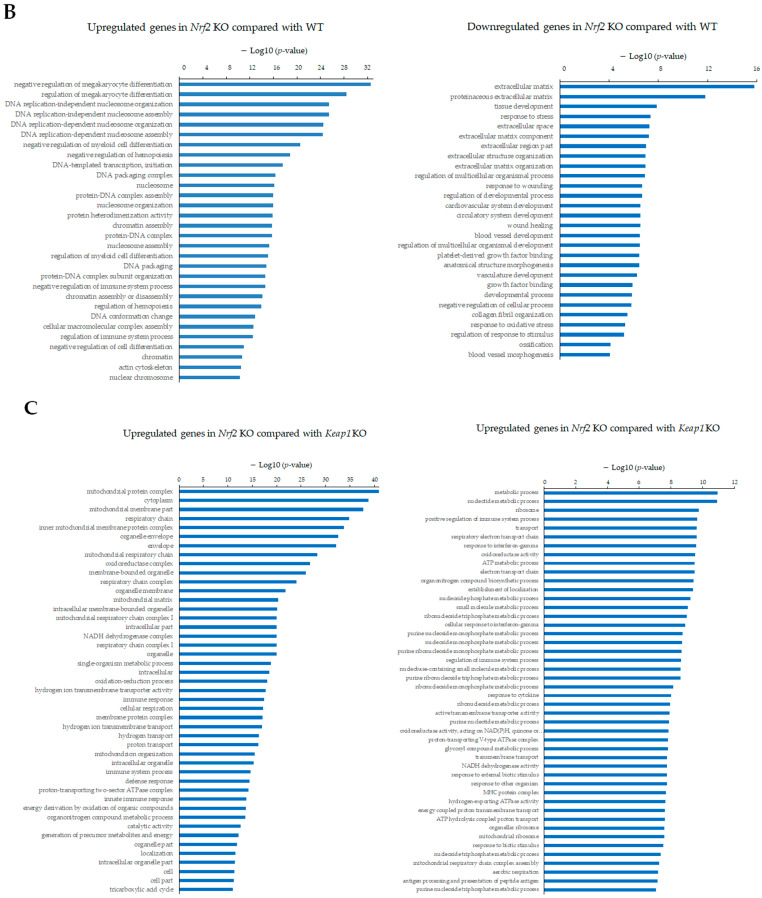

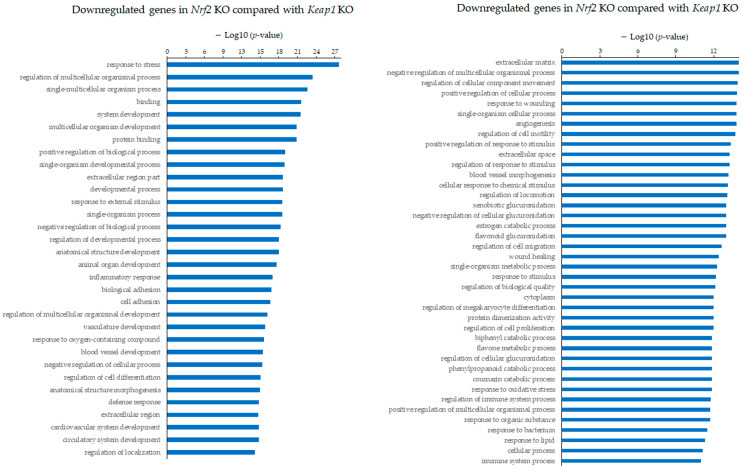
GO enrichment analysis. (**A**) Up- or downregulated genes were analyzed using the Database for Annotation, Visualization, and Integrated Discovery (DAVID) for GO enrichment analysis in *Keap1* KO cells compared with WT osteoclasts. (**B**) Up- or downregulated genes were analyzed using DAVID for GO enrichment analysis in *Nrf2* KO osteoclasts compared with WT osteoclasts. (**C**) Up- or downregulated genes were analyzed using DAVID for GO enrichment analysis in *Nrf2* KO osteoclasts compared with *Keap1* KO cells.

GO analysis, using the DAVID showed that upregulated genes identified in *Keap1* KO cells/WT osteoclasts were associated with the terms “response to stress”, “response to external stimulus”, “inflammatory response”, “response to bacterium”, and “defense response”, whereas downregulated genes were associated with the terms “mitochondrion”, “cytoplasmic part”, “cytoplasm”, “mitochondrial part”, and “mitochondrial inner membrane” (Figure 8A).

GO analysis showed that upregulated genes identified in *Nrf2* KO osteoclasts/WT osteoclasts were associated with the terms “negative regulation of megakaryocyte differentiation”, “regulation of megakaryocyte differentiation”, “DNA replication-independent nucleosome organization”, “DNA replication-independent nucleosome assembly”, and “DNA replication-dependent nucleosome organization”, whereas downregulated genes were associated with the terms “extracellular matrix”, “proteinaceous extracellular matrix”, “tissue development”, “response to stress”, and “extracellular space” (Figure 8B).

GO analysis showed that upregulated genes identified in *Nrf2* KO osteoclasts/*Keap1* KO cells were associated the with terms “mitochondrial protein complex”, “cytoplasm”, “mitochondrial membrane part”, “respiratory chain”, “inner mitochondrial membrane protein complex”, “organelle envelope”, “mitochondria respiratory chain”, and “oxidoreductase complex”, whereas downregulated genes were associated with the terms “response to stress”, “regulation of multicellular organismal process”, “single-multicellular organism process”, “binding”, and “system development” (Figure 8C).

### 3.3. KEGG Pathway Analysis of Nrf2/Keap1-Mediated Osteoclastogenesis

**Figure 9:**
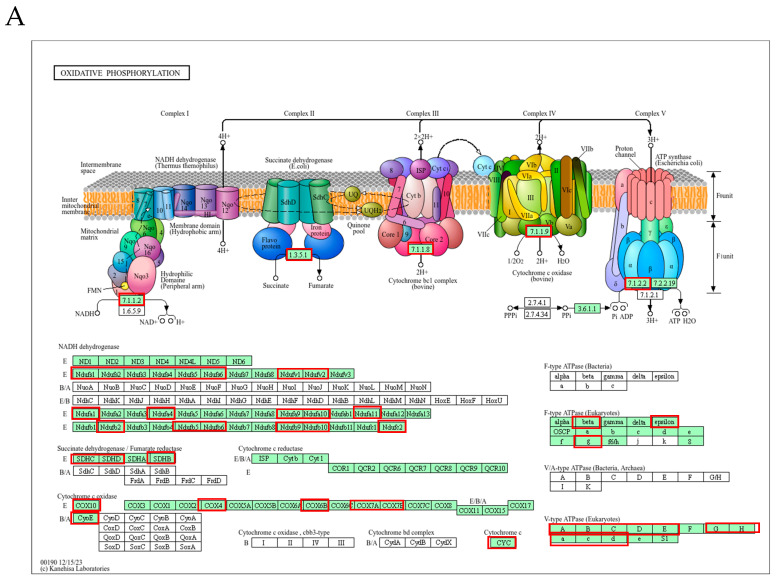

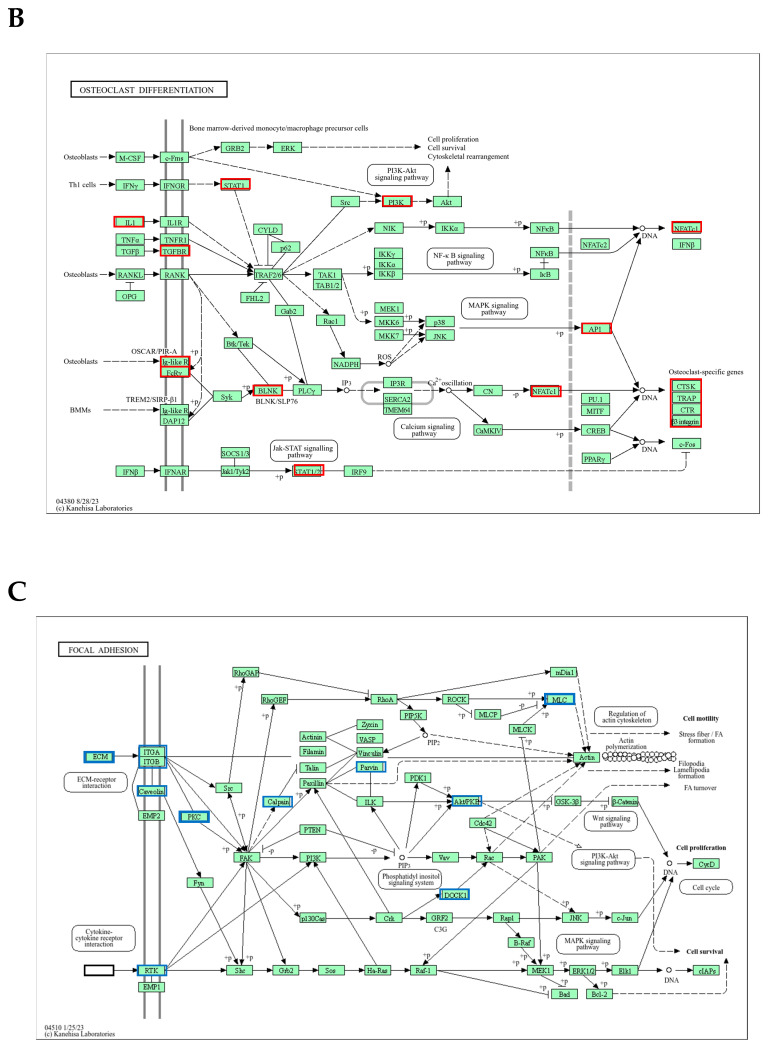

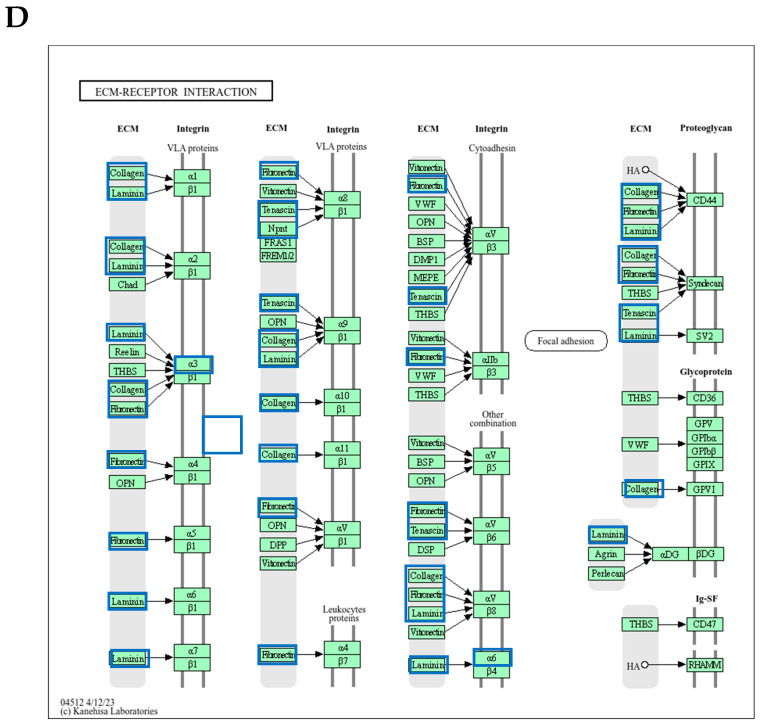
KEGG pathway enrichment analysis. Compared to *Keap1* KO cells, *Nrf2* KO osteoclasts exhibited a marked increase in expression of genes (surrounded by red lines) involved in oxidative phosphorylation (**A**) and osteoclast differentiation (**B**), whereas marked decreased in expression of genes (surrounded by blue lines) involved in focal adhesion (**C**) and ECM–receptor interaction (**D**).

The effect of the Nrf2/Keap1 system on osteoclastogenesis was also confirmed by KEGG analysis using DAVID for functional annotation of differentially expressed genes. Among the 683 upregulated genes with more than a 2-fold change in *Nrf2* KO osteoclasts compared with *Keap1* KO cells, 54 were associated with the oxidative phosphorylation pathway. Genes surrounded by red lines are significantly upregulated genes such as NADH:ubiquinone oxidoreductase core subunit (*Ndhfs*), cytochrome C oxidase (*Cox*), and succinate dehydrogenase complex (*Sdhc*) (Figure 9A). These genes also coincided with those whose expression was reduced in *Keap1* KO cells relative to WT osteoclasts.

Among the 683 upregulated genes with more than a 2-fold change in *Nrf2* KO osteoclasts than in *Keap1* KO cells, 13 were associated with the osteoclast differentiation pathway. Red asterisks indicate significantly upregulated genes, such as cathepsin K (*Ctsk*), calcitonin receptor (*Calcr*), and nuclear factor of activated T cells, cytoplasmic, calcineurin-dependent 1 (*Nfatc1*) (Figure 9B). These genes were also consistent with those that were downregulated in *Keap1* KO cells relative to WT osteoclasts. Moreover, 27 other genes, including integrin alpha V (*Itgav*), integrin beta3 (*Itgb3*), ATPase (*Atp6v*), and transporter 1 ATP-binding cassette (*Tap1*), whose expression is upregulated, are involved in the phagosome pathway.

Interestingly, among the 644 downregulated genes with less than a 0.5-fold change in *Nrf2* KO osteoclasts compared with *Keap1* KO cells, 24 genes were associated with the focal adhesion pathway. Blue asterisks indicate significantly decreased expression of genes such as caveolin-1 (*Cav1*), integrin alpha1 (*Itga1*), protein kinase C, beta (*Prkcb*), calpain2 (*Capn2*), pavin alpha (*Parva*), and dedicator of cytokinesis1 (*Dock1*) (Figure 9C).

Among 644 downregulated genes with less than a 0.5-fold change in *Nrf2* KO osteoclasts compared with *Keap1* KO cells, 16 genes were associated with the ECM-receptor interaction pathway. Genes surrounded by blue lines are significantly decreased expression of genes such as collagen, type1, alpha1 (*Col1a1*), laminin B1 (*Lamb1*), fibronectin1 (*Fn1*), tenascin (*Tnc*), and nephronectin (*Npnt*) (Figure 9D).

### 3.4. Protein–Protein Interaction Network

Interactions between proteins encoded by the upregulated genes in *Nrf2* KO osteoclasts relative to *Keap1* KO cells were predicted using GeneMANIA, the generated protein association network, in which proteins are presented in the form of nodes, and different colored lines between nodes represent specific meanings. Each color code within a node reflects its function. Of the 683 genes whose expression was upregulated in *Nrf2* KO osteoclasts, the nodes colored for mitochondria-related functions, such as the mitochondrial protein complex, mitochondrial inner membrane, respiratory chain complex, mitochondrial matrix, oxidative phosphorylation, mitochondrial transport, and mitochondrial gene expression, are shown in Supplementary Figure S1. Similarly, nodes colored for osteoclast-related functions, such as bone remodeling, response to tumor necrosis factor, bone resorption, macrophage migration, proton-transporting V-type ATPase complex, regulation of bone resorption, and regulation of osteoclast differentiation, are shown in Supplementary Figure S2.

**Figure 10:**
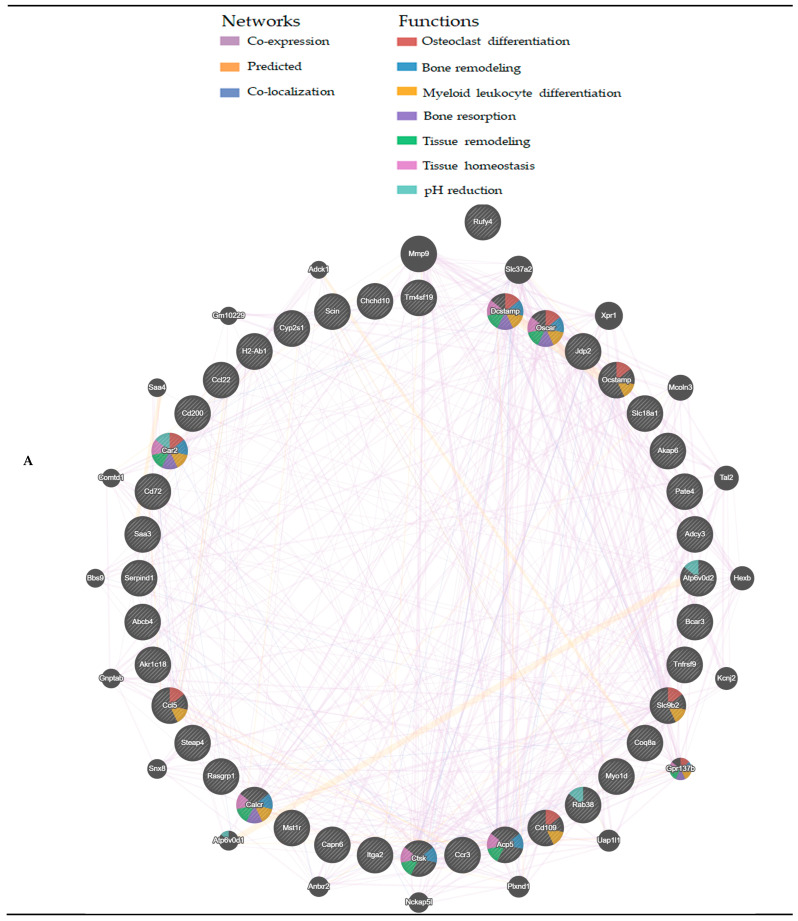

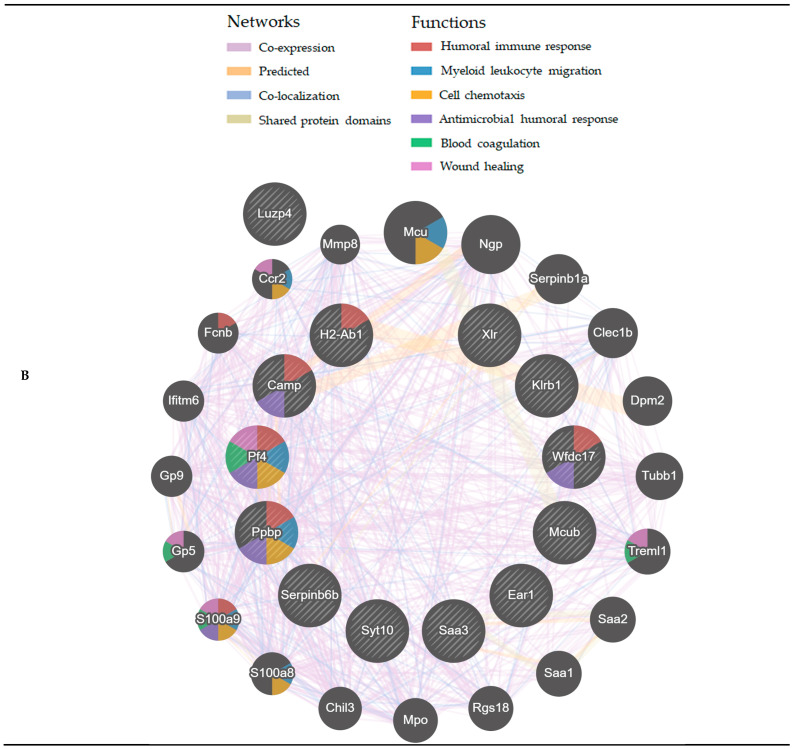
Protein–protein interaction network analysis by GeneMANIA. (**A**) Predicted network of proteins that interact with proteins encoded by the top 40 upregulated genes in *Nrf2* KO osteoclast against *Keap1* KO cells. (**B**) Predicted network of proteins interacting with proteins encoded by genes upregulated in Nrf2 KO osteoclast compared with WT osteoclasts.

Interactions between proteins encoded by the top 40 upregulated genes in *Nrf2* KO osteoclasts relative to *Keap1* KO cells were predicted using GeneMANIA, and the following four genes: mucolipin 3 (*Mcoln3*); T-cell acute lymphocytic leukemia 2 (*Tal2*); potassium-inward centrally rectifying channel, subfamily J, member 2 (*Kcnj2*); and plexin D1 (*Plxnd1*) were revealed. (Figure 10A). These four genes were not upregulated in the *Nrf2* KO cells in the present study. Among the top 40 genes, *Acp5*, *Adcy3*, *Atp6v0d2*, *Ctsk*, *Dcstamp*, and *Ocstamp* were predicted to be coexpressed with *Mcoln3*. Moreover, *Dcstamp*, *Jdp2*, *Mst1r*, *Serpind1*, and *Tnfrsf9* were predicted to be co-expressed with *Tal2*. *Acp5*, *Adcy3*, *Car2*, *Ccr3*, *Jdp2*, *Ocstamp*, and *Oscar* were predicted to be coexpressed with *Kcnj2*. Furthermore, *Acp5*, *Adcy3*, *Akap6*, *Car2*, *Ccr3*, *Ctsk*, *Cyp2s1*, *Dcstamp*, *Slc9b2*, *Jdp2*, and *Ocstamp* were predicted to be coexpressed with *Plxnd1* (Figure 10A). Moreover, some molecules were predicted to interact with the top 40 genes, such as matrix metallopeptidase 9 (*Mmp9*), solute carrier family 37 (*Slc37a2*), and G protein-coupled receptor 137 B (*Gpr137b*), which were significantly upregulated in *Nrf2* KO osteoclasts (Supplementary Table S6).

Among the top 40 genes, *Rufy4* was not predicted to interact with the other genes. Therefore, we reanalyzed the interaction between *Rufy4* and all other 682 genes whose expression was upregulated in *Nrf2* KO and predicted the interaction with arginine vasopressin-induced 1 (*Avpi1*), mitochondrial ribosomal protein L47 (*Mrpl47*), pleckstrin homology domain-containing family F (with FYVE domain) member 1 (*Plekhf1*), pleckstrin homology domain-containing family N member 1 (*Plekhn1*), and WD repeat and FYVE domain-containing 1 (*Wdfy1*). However, the functions of these genes in osteoclasts have not yet been reported.

As shown in Supplementary Table S4, no genes were upregulated more than 4-fold in *Nrf2* KO osteoclasts relative to WT osteoclasts, likely because osteoclasts form in both *Nrf2* KO and WT cells (Figure 1A). *Nrf2* KO cells exhibit enhanced osteoclastogenesis and bone resorption compared to WT cells [21]. The genes that were upregulated in the current microarray suggested that they contributed to enhanced osteoclastogenesis in *Nrf2* KO conditions. GeneMANIA predicted interactions between the 24 genes whose expression was upregulated more than 2-fold in *Nrf2* KO cells and predicted that WAP four-disulfide core domain 17 (Wfdc17), histocompatibility 2, class II antigen A, beta1 (H2-Ab1), and platelet factor 4 (Pf4) interact with mitochondrial calcium uniporter dominant negative beta subunit (Mcub), triggering receptor expressed on myeloid cells-like 1 (Treml1), S100 calcium protein A9 (S100a9), and chemokine (C-C motif) receptor 2 (Ccr2), which are not included in the 24 genes (Figure 10B). These molecules are involved in humoral immune response, myeloid leukocyte migration, cell chemotaxis, blood coagulation, and wound healing, suggesting that these functions might effectively induce osteoclastogenesis. Among the 24 genes, leucine zipper protein 4 (Luzp4) showed no interaction with the other genes (Figure 10B).

## 4. Discussion

In this study, we used WT, *Nrf2* KO, and *Keap1* KO neonatal mouse spleen cells as osteoclast progenitor cells and performed DNA microarray analysis of the RNA obtained from the cells three days after RANKL addition. The results obtained by PCA are shown in Supplementary Tables S2–S7. Using these data, we performed GO enrichment analysis, KEGG pathway analysis, and constructed a protein–protein interaction network using GeneMANIA. Although various factors have been reported to regulate osteoclast differentiation, this study focused on Nrf2 and Keap1, which are essential for oxidative stress regulation, and comprehensively investigated genes whose expression was upregulated during RANKL-induced osteoclast differentiation.

In this study, *Nrf2* KO cells with accelerated osteoclast differentiation compared with *Keap1* KO cells showed significant upregulation of mitochondria-related genes, including cytochrome C oxidase (*Cox*), mitochondrial ribosomal protein 12 (*Mrpl12*), and succinate dehydrogenase complex assembly factor 1 (*Sdhaf1*), as well as osteoclast differentiation marker genes, including carbonic anhydrase2 (*Car2*), integrin beta3 (*Itgb3*), osteoclast-associated receptor (*Oscar*), and dendrocyte-expressed seven transmembrane protein (*Dcstamp*). These results suggest that mitochondrial biogenesis was induced in *Nrf2* KO osteoclasts.

Previous studies have shown that cells and mitochondria isolated from *Nrf2* KO mice have low respiration and ATP levels, whereas *Keap1* knockout and knockdown mice have increased respiration and ATP levels [26,27]. Furthermore, mitochondrial fatty acid oxidation was decreased in mitochondria isolated from *Nrf2*-deficient mice, indicating that Nrf2 positively regulates mitochondrial biogenesis [28]. However, these results are inconsistent with our findings of elevated expression of mitochondria-related genes in *Nrf2* KO osteoclasts. The increased expression of peroxisome proliferative-activated receptor gamma coactivator 1-beta (*Ppargc1b*) [29] in *Nrf2* KO osteoclasts compared to that in Keap1 KO cells in the current study suggests that ROS, increased by *Nrf2* KO, induced the phosphorylation of CREB and increased Ppargc1b, possibly resulting in increased mitochondrial biogenesis. Alternatively, because osteoclasts are mitochondria-rich cells, the comparison of *Nrf2* KO, which promotes osteoclast formation, with *Keap1* KO, in which only a few osteoclasts are formed, may have resulted in the higher expression of mitochondria-related genes, reflecting prominent osteoclast characteristics.

Microarray analysis has led to the identification of genes with unknown functions in osteoclasts. Our microarray results showed that the expression of Rufy4, a novel autophagy regulator, was higher in *Nrf2* KO osteoclasts. We also found that Rufy4 expression was upregulated during RANKL-induced osteoclast differentiation and that bone resorption was enhanced in osteoclasts overexpressing Rufy4 [30]. Kim et al. reported that Rufy4 promotes extracellular secretion of cathepsin K and increases bone resorption [31].

Moreover, qRT-PCR results showed that the gene expression of *Snhg6*, Ccdc109b, *Wfdc17*, and *Ppbp* was upregulated in *Nrf2* KO osteoclasts compared with WT osteoclasts, but the roles of these genes in osteoclasts have not been clarified. However, the present GeneMANIA analysis predicted *Mcub*, *Treml1*, *S100a9*, and *Ccr2* as genes that interact with Wfdc17 and Ppbp through protein–protein interactions. MCUb is the inhibitory subunit of the MCU complex. MCU is a mitochondrial inner membrane protein that transports cytosolic calcium into the mitochondrial matrix and reduces intracellular oxidative phosphorylation levels in *Mcu*-KO mice [32]. The MCU inhibitor ruthenium red has been reported to inhibit RANKL-induced osteoclast differentiation and bone resorption [33]. Treml1 has been reported to be a negative regulator of osteoclastogenesis [34]. S100a9 repressed RANK expression and inhibited monocyte differentiation into osteoclasts. Ccr2 and its ligand Ccl2 have been reported to promote osteoclastogenesis [35]. Wfdc17 and Ppbp have been suggested to function in osteoclasts via these genes.

Bone metabolism is closely related to oxidative stress, and the Nrf2/Keap1 signaling axis regulates osteoclastogenesis. Therefore, bioactive natural compounds are considered to have potential Nrf2-activating effects and to reduce the risk of oxidative stress-related osteoporosis [22]. Recent studies have reported that plant phenolic compounds such as 4-methylcatechol [36], oroxylin A [37], notopterol [38], and tussilagone [39] have inhibitory effects on osteoporosis by acting on the Nrf2/Keap1 signaling axis and reducing oxidative stress levels. It is unclear whether these natural compounds are involved in the expression of the genes identified in this study; however, further studies are expected in the future.

The lack of Nrf2 can induce osteoclastogenesis; however, the role of Nrf2 in osteoblast differentiation is more complex. Our previous study revealed that during osteoblast differentiation using primary osteoblasts isolated from newborn mice calvaria cultured with ascorbic acid, β-glycerophosphate, and dexamethasone, expression levels of osteogenic essential genes such as Runx2, Sp7, and Alpl were significantly upregulated in *Nrf2* KO cells. In contrast, the expression of these genes was suppressed by approximately 50% in *Keap1* KO cells compared to WT cells. In co-culture experiments with primary osteoblasts from newborn mice and splenic macrophages, *Keap1* KO macrophages show completely abolished osteoclastogenesis; however, *Keap1* KO osteoblasts could support osteoclastogenesis. This is probably due to the fact that the expression of the osteogenic essential genes is not completely suppressed but is expressed even in half levels of the WT cells [21]. Consistent with our results, Park et al. reported that the gene expression of ALP, Runx2, and Sp7 was significantly upregulated in *Nrf2* KO osteoblasts [40]. In other reports, *Nrf2* KO mice showed lower bone mass and decreased bone formation rate [41], and *Nrf2*-deficient osteoblasts exhibited impaired differentiation and mineralization [42]. The role of Nrf2 in osteoblast differentiation and osteogenesis remains controversial. It may depend on the age of the mice and the level of oxidative stress in each experimental system. Loss of Keap1 promotes hyperactivation of Nrf2, resulting in juvenile lethality. Therefore, it was not possible to observe hard tissues of adult individuals of global *Keap1* KO mice. Yoshida et al. used viable *Keap1* KO mice by esophageal *Nrf2* deletion in *Keap1* KO mice and revealed impaired differentiation of both osteoclasts and osteoblasts [43]. These studies suggest that the Nrf2/Keap1 signaling axis plays an important role in osteoblasts as well as osteoclastogenesis.

## 5. Conclusions

The results of the current DNA microarray transcriptome analysis revealed elevated expression of novel genes, such as *Rufy4*, *Avpi1*, *Mrpl47*, *Wfdc17*, and *H2-Ab1*, in *Nrf2* KO osteoclasts. It is expected that the data from this study will be further analyzed by researchers for their own purposes, resulting in the elucidation of a new regulatory mechanism of osteoclast differentiation and the development of therapeutic agents for diseases caused by excessive bone resorption by osteoclasts, such as osteoporosis, rheumatoid arthritis, and periodontal diseases.

## Figures and Tables

**Table 1:** Top 20 upregulated genes in *Keap1* KO/WT.

Gene Symbol	Description	Fold Change (log)	Fold Change
*Nqo1*	NAD(P)H dehydrogenase, quinone1	4.539824	23.260722
*Il1f9*	Interleukin 1 family, member 9	3.949011	15.444399
*A5* *30064D06Rik*	RIKENcDNAA530064D06gene	3.487380	11.215177
*Mmp12*	Matrix metallopeptidase 12	3.240210	9.449317
*Slc39a4*	Solute carrierfamily39, member 4	3.164959	8.969074
*Fabp7*	Fatty acid binding protein7, brain	3.153433	8.897708
*Cxcl14*	Chemokine (C-X-C motif) ligand14	3.124515	8.721130
*Gsta3*	Glutathione S-transferase, alpha3	3.060010	8.339784
*Prl2c2*	Prolactinfamily2, subfamily c	2.981902	7.900270
*Rnf128*	Ring finger protein 128	2.961491	7.789289
*Treml4*	Triggering receptor expressed on myeloid cells-like 4	2.820159	7.062402
*Ly6g*	Lymphocyte antigen 6 complex, locus G	2.7996016	6.962482
*Car3*	Carbonic anhydrase 3	2.7337945	6.652029
*Tanc2*	Tetra tri co peptide repeat	2.7162542	6.571643
*Gclm*	Glutamate-cysteine ligase, modifier subunit	2.683442	6.423867
*Cd36*	CD36antigen	2.649393	6.274032
*Ceacam10*	Carcinoembryonic antigen-related cell adhesion molecule 10	2.6434088	6.248062
*Gsta4*	Glutathione S-transferase, alpha 4	2.447667	5.455330
*Mfsd6*	Major facilitator superfamily domain containing 6	2.433969	5.403780
*Itga8*	Integrin alpha 8	2.415440	5.334820

**Table 2:** Bottom 20 downregulated genes in *Keap1* KO/WT.

Gene Symbol	Description	Fold Change (log)	Fold Change
*Car2*	Carbonic anhydrase 2	−6.374898	0.012049
*Calcr*	Calcitonin receptor	−6.056974	0.015020
*Oscar*	Osteoclast associated receptor	−5.767575	0.018356
*Scin*	Scinderin	−5.017828	0.030866
*Ctsk*	Cathepsin K	−4.974964	0.031797
*Akr1c18*	Aldo-keto reductase family 1, member C18	−4.904193	0.033396
*Pate4*	Prostate and testis expressed 4	−4.870079	0.034195
*Ocstamp*	Osteoclast stimulatory transmembrane protein	−4.830698	0.035141
*Ccr3*	Chemokine (C-C motif) receptor 3	−4.745497	0.037279
*Tm4sf19*	Transmembrane 4L six family member 19	−4.693895	0.038636
*Slc9b2*	Solute carrier family 9, subfamily B	−4.684105	0.038899
*Akap6*	A kinase (PRKA) anchor protein 6	−4.575950	0.041928
*Steap4*	STEAP family member 4	−4.502150	0.044128
*Cd200*	CD200 antigen	−4.479137	0.044838
*Dcstamp*	Dendrocyte expressed seven transmembrane protein	−4.210025	0.054033
*Trav9d-3*	T cell receptor alpha variable 9D-3	−4.199091	0.054444
*Atp6v0d2*	ATPase, H+ transporting, lysosomal V0 subunit D2	−4.196109	0.054556
*Myo1d*	Myosin I D	−4.178478	0.055227
*Adck3*	Aar F domain containing kinase 3	−3.985752	0.063120
*Rasgrp1*	RAS guanyl releasing protein 1	−3.979324	0.063402

**Table 3:** Top 20 upregulated genes in *Nrf2* KO/WT.

Gene Symbol	Description	Fold Change (log)	Fold Change
*Snhg6*	Small nucleolar RNA host gene 6	1.870724	3.657160
*Ccdc109b*	Coiled-coil domain containing 109 B	1.842320	3.585861
*Wfdc17*	WAP four-disulfide core domain 17	1.541663	2.911298
*Mir1945*	Micro RNA 1945	1.474677	2.779214
*Ear1|Ear-ps9*	Eosinophil-associated, ribonuclease A family, member1	1.378982	2.600848
*1700049L16Rik*	Hematological and neurological expressed 1-like pseudogene	1.346564	2.543057
*H2-Q6|H2-Q8*	Histocompatibility 2, Q region locus 6	1.3387494	2.529320
*Fam136b-ps*	Family with sequence similarity136, member B, pseudogene	1.301304	2.464516
*H2-Ab1*	Histocompatibility 2, classII antigen A, beta1	1.263337	2.400504
*Igh-VJ558*	Immunoglobulin heavy chain (J558 family)	1.254574	2.385967
*Pf4*	Pro-platelet basic protein	1.177933	2.262523
*Luzp4*	Leucine zipper protein 4	1.128823	2.186803
*Ppbp*	Pro-platelet basic protein	1.121270	2.175384
*Mir3093*	microRNA3093	1.114681	2.165472
*Trbc1|Tcrb-J*	T cell receptor beta, constant region 1	1.100635	2.144490
*Syt10*	Synaptotagmin X	1.081524	2.116271
*Tcrg-V4*	T cell receptor gamma, variable4	1.050665	2.104195
*Saa3*	Serum amyloid A3	1.050665	2.071484
*Klrb1*	Killer cell lectin-like receptor subfamily B member 1	1.031235	2.043773
*Serpinb6b*	Serine (or cysteine) peptidase inhibitor, clade B, member 6b	1.021454	2.029964

**Table 4:** Bottom 20 downregulated genes in *Nrf2* KO/WT.

Gene Symbol	Description	Fold Change (log)	Fold Change
*Ctse*	Cathepsin E	−3.9513383	0.064644
*Ifi202b*	Interferon activated gene 202B	−3.260101	0.104379
*Me1*	Malic enzyme 1, NADP (+)-dependent, cytosolic	−3.171412	0.110997
*Cbr3*	Carbonyl reductase 3	−2.686977	0.155289
*Bgn*	Biglycan	−2.376685	0.192551
*Thy1*	Thymus cell antigen 1, theta	−2.071330	0.237940
*Ppp2r1b*	Protein phosphatase 2, regulatory subunit A	−2.040244	0.243123
*Lrrc32*	Leucine rich repeat containing 32	−2.038478	0.243420
*Rnf128*	Ring finger protein 128	−2.035712	0.243888
*Ednrb*	Endothelin receptor type B	−1.992971	0.251221
*Cxcl14*	Chemokine (C-X-C motif) ligand 14	−1.991431	0.251489
*Loxl1*	Lysyl oxidase-like 1	−1.920795	0.264109
*Slc7a11*	Solute carrier family 7	−1.908276	0.266411
*Serpine1*	Serine (or cysteine) peptidase inhibitor	−1.894347	0.268995
*Cnn1*	Calponin 1	−1.877003	0.272249
*Ddah1*	Dimethyl arginine dimethyl amino hydrolase 1	−1.857229	0.276006
*Des*	Desmin	−1.813092	0.284580
*Pydc3*	Pyrin domain containing protein 3	−1.807523	0.285681
*Ctgf*	Connective tissue growth factor	−1.790487	0.289074

**Table 5:** The increased top 20 genes in *Nrf2* KO/*Keap1* KO.

Gene Symbol	Description	Fold Change (log)	Fold Change
*Car2*	Carbonic anhydrase 2	6.611182	97.760632
*Calcr*	Calcitonin receptor	5.738293	53.382416
*Pate4*	Prostate and testis expressed 4	5.416601	42.712931
*Oscar*	Osteoclast associated receptor	5.297467	39.327512
*Scin*	Scinderin	5.254921	38.184653
*Akr1c18*	Aldo-keto reductase family 1, member C18	5.221919	37.321084
*Ctsk*	Cathepsin K	5.070246	33.596662
*Ocstamp*	Osteoclast stimulatory transmembrane protein	4.729692	26.532560
*Slc9b2*	Solute carrier family 9, subfamily B	4.721196	26.376764
*Cd200*	CD200 antigen	4.569846	23.749842
*Akap6*	A kinase (PRKA) anchor protein 6	4.497392	22.586545
*Steap4*	STEAP family member 4	4.302776	19.736254
*Adck3*	aarF domain containing kinase 3	4.216301	18.588011
*Dcstamp*	Dentrocyte expressed seven transmembrane protein	4.199798	18.376600
*Tm4sf19*	Transmembrane 4L six family member 19	4.109665	17.263643
*Atp6v0d2*	ATPase, H+ transporting, lysosomal V0 subunit D2	4.063965	16.725356
*Rasgrp1*	RAS guanyl releasing protein 1	3.950285	15.458039
*Ccr* *3*	Chemokine (C-Cmotif) receptor 3	3.859836	14.518659
*Trav9d-3*	T cell receptor alpha variable 9D-3	3.780108	13.738075

**Table 6:** Bottom 20 downregulated genes in *Nrf2* KO/*Keap1* KO.

Gene Symbol	Description	Fold Change (log)	Fold Change
*Nqo1*	NAD(P)H dehydrogenase, quinone 1	−6.131591	0.014263
*Ctse*	Cathepsin E	−5.380067	0.024013
*Cxcl14*	Chemokine (C-X-C motif) ligand 14	−5.115946	0.028837
*Rnf128*	Ring finger protein 128	−4.997204	0.031311
*Me1*	Malic enzyme 1, NADP (+)-dependent,cytosolic	−4.568336	0.042150
*Mmp12*	Matrix metallo peptidase 12	−4.524167	0.043460
*Slc39a4*	Solute carrier family 39(zinc transporter)	−4.320142	0.050062
*Ednrb*	Endothelin receptor type B	−4.040073	0.060788
*Gclm*	Glutamate-cysteine ligase, modifier subunit	−3.970209	0.063804
*Tanc2*	Tetra tri copeptide repeat	−3.957151	0.064384
*Slc7a11*	Solute carrier family 7, member 11	−1.991431	0.251489
*Cbr3*	Carbonyl reductase 3	−3.728080	0.075463
*Gatm*	Glycine amidino transferase	−3.394802	0.095074
*Fabp7*	Fatty acid binding protein 7, brain	−3.334516	0.099131
*LOC100038947*	Signal-regulatory protein beta1-like	−3.313690	0.100573
*Nlrp1c-ps*	NLR family, pyrin domain containing 1C, pseudogene	−3.270247	0.103647
*Lrrc32*	Leucine rich repeat containing 32	−3.263585	0.104127
*Gsta3*	Glutathione S-transferase, alpha 3	−3.252826	0.104906
*Gsta4*	Glutathione S-transferase, alpha 4	−3.232622	0.106386

## Data Availability

The original contributions of this study are included in the article and Supplementary Materials. All the microarray datasets described in this paper are included and available in the article/Supplementary Materials. Further inquiries should be made with the corresponding author (E.S.).

## Supplementary Materials

The following supporting information can be downloaded at https://www.mdpi.com/article/10.3390/antiox13121575/s1: Figure S1: Protein–protein interaction network analysis by GeneMANIA; Figure S2: Protein–protein interaction network analysis by GeneMANIA; Table S1: List of QPCR primers; Table S2: Upregulated genes in Keap1 KO compared with WT; Table S3: Downregulated genes in Keap1 KO compared with WT; Table S4: Upregulated genes in Nrf2 KO compared with WT; Table S5: Downregulated genes in Nrf2 KO compared with WT; Table S6: Upregulated genes in Nrf2 KO compared with Keap1 KO; and Table S7: Downregulated genes in Nrf2 KO compared with Keap1 KO.
