# Supplementary material for: Transcriptomic Characterization Reveals Mitochondrial Involvement in Nrf2/Keap1-Mediated Osteoclastogenesis
Source: Antioxidants (Basel). 2024 Dec 20;13(12):1575. doi: 10.3390/antiox13121575 (PMC11673794; doi:10.3390/antiox13121575)
Supplement: Supplementary file 1 [file antioxidants-13-01575-s001.zip › Supplementary Table S1_Figure S1_S2.pdf]

Supplementary Materials

Transcriptomic characterization reveals mitochondrial involvement in Nrf2/Keap1-mediated osteoclastogenesis

Eiko Sakai and Takayuki Tsukuba

Gene symbol	**Forward**	**Reverse**
β-actin	acccagatcatgtttgagac	gtcaggatcttcatgaggtagt
Nqo1	aagagctttagggtcgtcttggca	agcctccttcatggcgtagttgaa
Il1f9	acagagtaaccccagtcagc	gtccgggtgtggtaaaacag
Mmp12	gctgctcccatgaatgacag	tgccagagttgagttgtcca
Slc39a4	agcaatctccgacagtccaa	cagctcatgacagaacaccg
Fabp7	gtcaggaaggtggcaaagtg	gtcacgaccatcttgccatc
Cxcl14	ctacagcgacgtgaagaagc	cgttccaggcattgtaccac
Gsta3	tgcccatggtagagatcgac	ttgcctctttctcctcaggg
Rnf128	gcaaccgtgggctatttcat	ggttaggatgcgcaccaaat
Ly6g	gggctggagtgctacaattg	gcagatgggaaggcagagat
Car3	tcctctctctggaccctacc	aaaatgccaaccacagcgat
Tanc2	tcctcctcaacctctctcga	gtctgcactcttcctccact
Gclm	tcagccccgatttagtcagg	tgccatgtcaactgcacttc
Calcr	cgcatccgcttgaatgtg	tctgtctttccccaggaaatga
Scin	agatgcaagtgggtccatga	cagtcttcctctcctgtggg
Ctsk	cagcttccccaagatgtgat	agcaccaacgagaggagaaa
Pate4	ttgtggagggtctgatctgt	cactgaggcgagcacatttg
Ocstamp	tgggcctccatatgacctcgagtag	tcaaaggcttgtaaattggaggagt
Ccr3	ttttcctgcagtcctcgcta	taagacggatggccttgtgt
Tm4sf19	atcgcgctcctctttcctaa	aagcaaatcacagccccaag
Steap4	cattgctcgtactcttgggc	gattcgggatggaaatggcc
Snhg6	acaagctgccaggactacat	tgactttgttcatggccttca
Ccdc109b	aaactgcagccatcatcacg	cgctctcgtctcttctggat
Wfdc17	gtggctttgatcactgtggg	tgcagacatgaccacagcta
Ppbp	tcagacctacatcgtcctgc	agtgaactcctggcctgtac
Ctse	gccagacctttgtgaatgca	cagttgaggctcccagagaa
Ifi202b	aagtggtggcattgaaagca	tggctcttcacctcagacac
Me1	ggatatggctgccttcaacg	aactccagggaacacgtagg
Cbr3	tctgcactgagttactgcct	ccaaccttccctctcatgga
Thy1	tgaaccaaaaccttcgcctg	agtagtcgccctcatccttg
Lrrc32	ccctggaggtgctagatctg	caaagcggcagattaggtcc
Slc7a11	gtctgcctgtggagtactgt	attacgagcagttccaccca
Oscar	ctgctggtaacggatcagctccccaga	ccaaggagccagaaccttcgaaact
Akr1c18	aagtgtaaggatgcagggct	atctttgagttcccagggca
Adck3	tgttccctgagcacctgatt	ttcctcacttcctggctcag
Atp6v0d2	ttcatctccagaccacggac	tcttttgcaaggccccattc
Rasgrp1	gaagcgaatcaagagcccag	tccttgctctcctcactgtg

Supplementary TableS1: List of QPCR primers

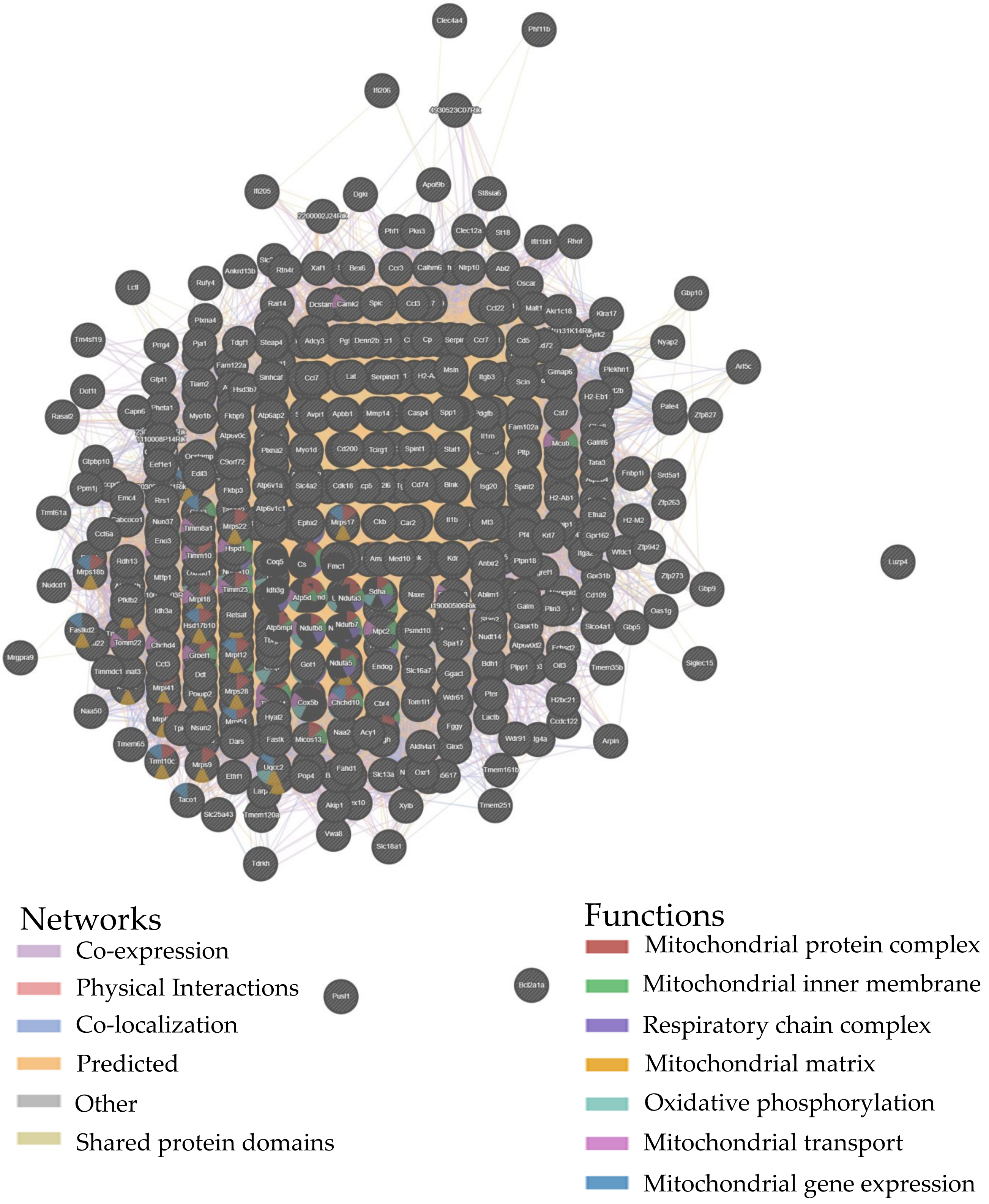

Supplementary Figure S1: Protein-protein interaction network analysis by GeneMANIA. Predicted network of proteins that interact with proteins encoded by 683 genes upregulated by Nrf2 KO osteoclast against Keap1 KO cells. Genes with mitochondria-related functions are colored.

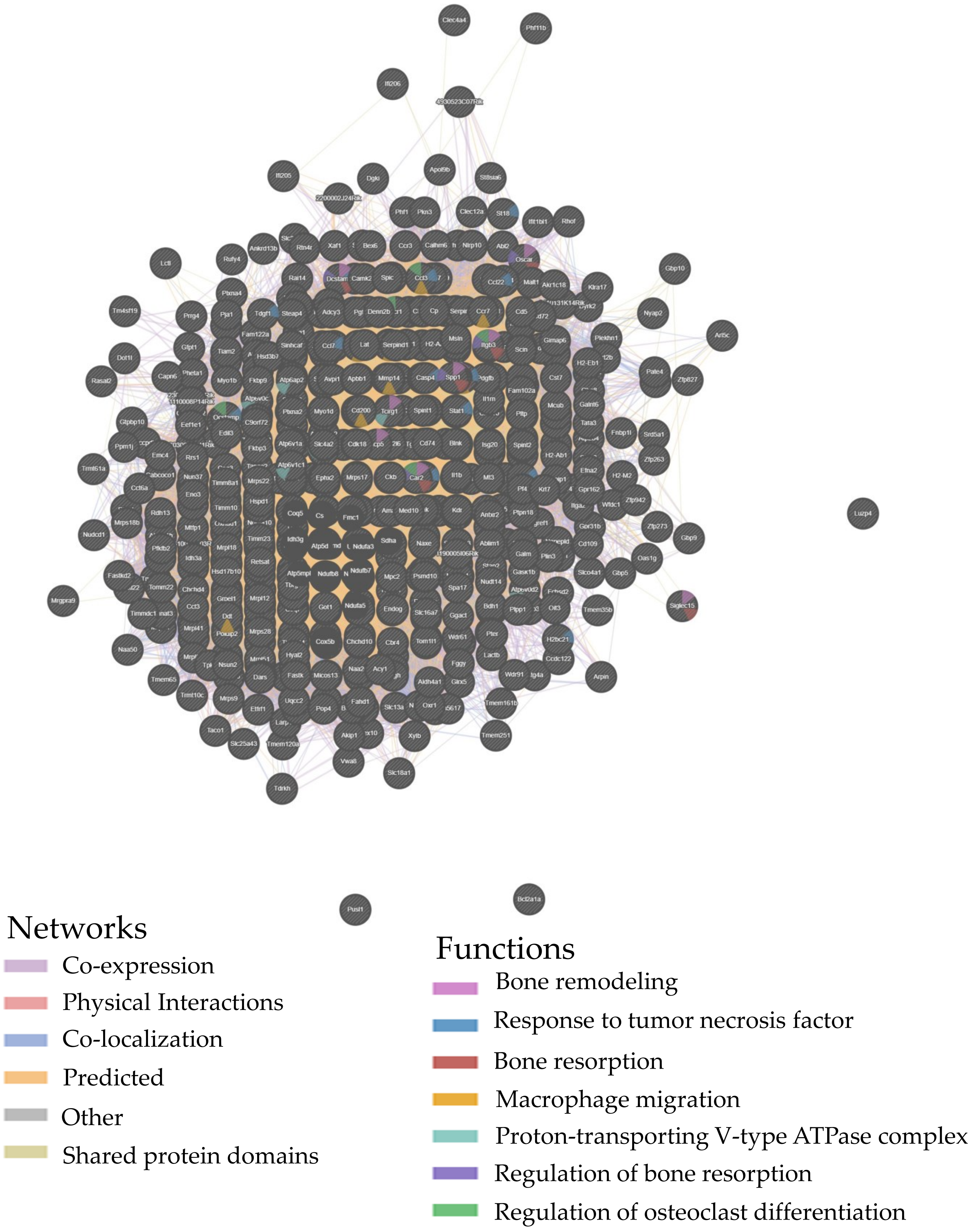

Supplementary Figure S2: Protein-protein interaction network analysis by GeneMANIA. Predicted network of proteins that interact with proteins encoded by 683 genes upregulated by Nrf2 KO osteoclast against Keap1 KO cells. Genes with osteoclast-related functions are colored.
